# Neuropathic Bladder Caused by Caudal Regression Syndrome without Any Other Neurogenic Symptoms

**DOI:** 10.1155/2012/982418

**Published:** 2012-06-12

**Authors:** Yoshinobu Moritoki, Yoshiyuki Kojima, Hideyuki Kamisawa, Kentaro Mizuno, Kenjiro Kohri, Yutaro Hayashi

**Affiliations:** Department of Nephro-Urology, Nagoya City University Graduate School of Medical Sciences, Nagoya 467-8601, Japan

## Abstract

Caudal regression syndrome (CRS) is a rare congenital vertebral anomaly, which occurs most often in combination with spinal cord malformations and morphologic dysfunctions of the lower extremities; these signs are useful for both patients and clinicians in the diagnosis of this syndrome. However, in certain cases, clinicians have failed to identify the syndrome due to the lack of apparent anomalies, resulting in the progression of renal dysfunction caused by neuropathic bladder when CRS is eventually identified. Here, we report a case of a 2-year-old girl who was referred to our hospital for vesicoureteral reflux. At examination, she presented no neurological symptoms; however, on cystourethrography and CT scanning we found that the sacral bone was absent, through which CRS was diagnosed. A urodynamic study indicated detrusor-sphincter dyssynergia, and clean intermittent catheterization was initiated. In the present report, we describe a case of CRS with no neurologic symptoms other than a neuropathic bladder. The lack of outward signs can result in delayed diagnosis. Thus, urological examinations, including a urodynamic study, might be the only clue for identifying an underlying neurologic injury involving the lower spinal cord.

## 1. Introduction

 Caudal regression syndrome (CRS) is a rare congenital anomaly characterized by caudal vertebral agenesis or dysgenesis, most often in combination with spinal cord malformations [1], with an estimated incidence of approximately 0.1 to 0.25 per 10,000 births [2]. Patients with CRS are first investigated for neurologic, orthopedic, and urologic complaints [1]. In general, neurogenic or orthopedic dysfunction suggesting CRS is detected earlier than urologic dysfunction, because these apparent impairments are easier to identify. Unless patients have morphologic abnormalities or urinary tract infections (UTIs), at times, parents fail to notice their child's urologic disorder, and renal impairment can develop. As a result, these children frequently have progressive kidney damage when CRS is identified. Here we report a case of neuropathic bladder caused by CRS without any neurogenic symptoms and discuss the role of urologic examinations and interventions.

## 2. Case Presentation

A two-year-old girl was referred to the Urology Department for the management of vesicoureteral reflux (VUR). She had initially had a urinary tract infection (UTI) before presentation at the age of two, when right hydronephrosis and VUR on the ipsilateral side (Grade IV) were noted. She was the offspring of nonconsanguineous parents and was a twin, born at 29 weeks' gestation, weighing 2,069 g. Antenatal ultrasound had not exhibited any abnormalities, and at birth she had no deformities of the limbs or anus. There was no significant past history. Her mother was not diabetic. 

On examination, she was active and alert, in satisfactory general health, and had passed normal mental milestones. She had a normal lower region and buttocks, no natal cleft, and no dimples at the hips and knees. Equinovarus deformity was slightly present, but had not been identified on previous medical checkups. She was able to walk and move her lower limbs, had sensations in the lower limbs and perineum, and had normal tendon refluxes, including the anocutaneous reflex. She had no anal stenosis or deformity. She had no constant urine leakage, but had urine dribble during emptying; however, her parents had failed to recognize this as abnormal. Other systems were clinically normal.

**Figure 1:**
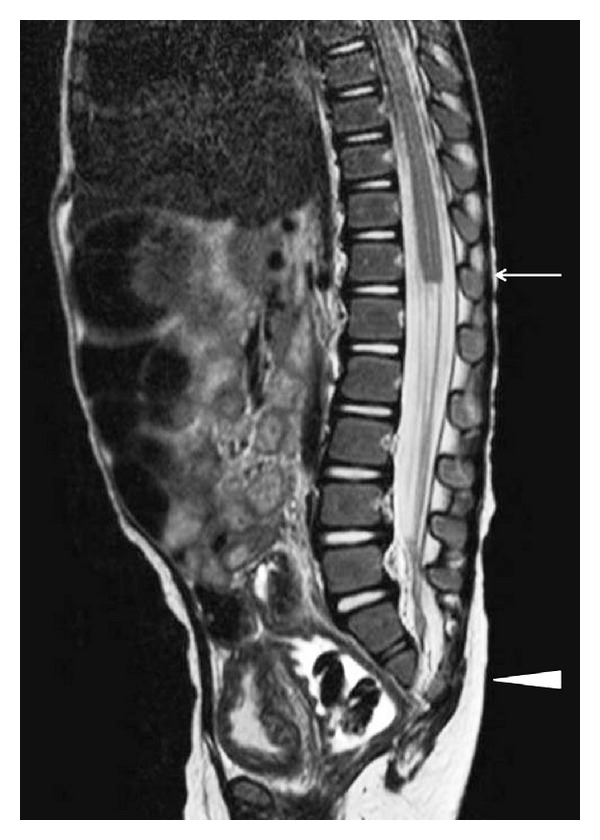
MRI showed the absence of the sacral bone under S2 and distal lumbar vertebrae (arrowhead). The level of the spinal cord terminus was situated as T-12 (arrow).

**Figure 2:**
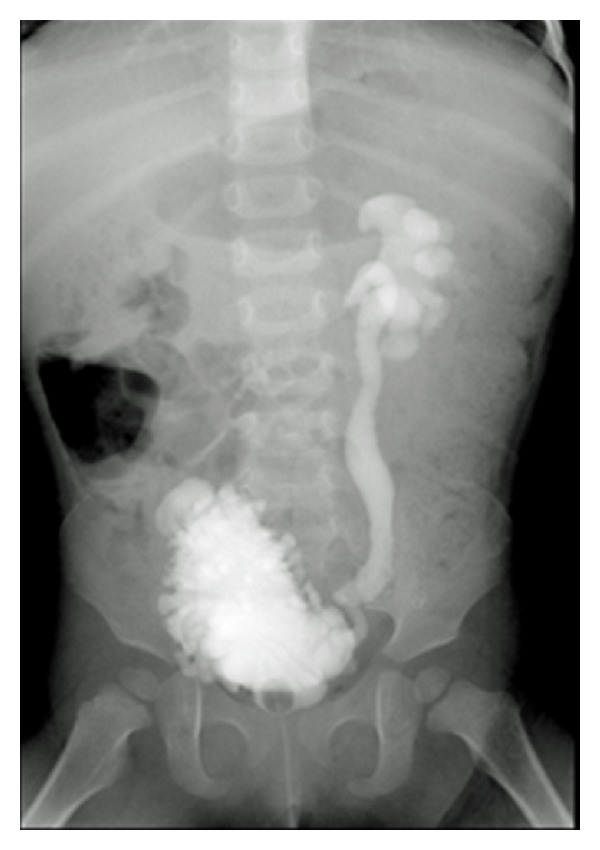
Cystogram showed a trabeculated bladder with fairly poor capacity and grade IV reflux.

**Figure 3:**
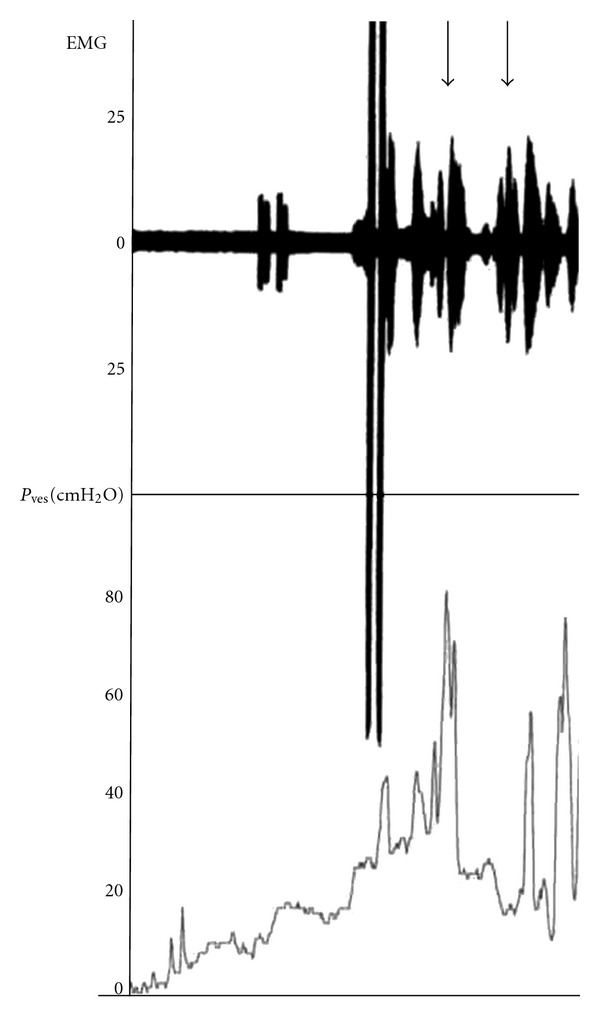
Electromyography (EMG) showed impaired compliance with detrusor overactivity associated with low-volume urine. Increased sphincter activity consistent with detrusor sphincter dyssynergia (arrow). Detrusor leak point pressure was 50 cmH_2_O, and maximum intravesical pressure was 80 cmH_2_O. Maximum bladder capacity was 170 mL with no leakage.

Urinalysis showed pyuria without bacteria. Ultrasound revealed bilateral hydronephrosis of grade III by The Society for Fetal Urology guidelines. X-ray and CT of the lower region showed the absence of the sacral bone under S2 and distal lumbar vertebrae. The limb bones were not hypoplastic. On MRI T1- and T2-weighted sagittal images of the lumbosacral spine showed partial agenesis of the sacrum, a truncated cord and filum terminale (Figure 1). A cystogram taken at another hospital showed a trabeculated bladder with fairly poor capacity and with grade IV reflux (Figure 2). Urodynamic study showed sphincter-detrusor dyssynergia (DSD) with maximum bladder pressure of 80 cmH_2_O (Figure 3). The maximum bladder capacity was 170 mL with no leakage. DMSA showed renal scarring on both kidneys, suggesting kidney damage, which was thought to have been caused by the neuropathic bladder and VUR. The diagnosis of caudal regression syndrome was made, and clean intermittent catheterization (CIC) by her parent was started. 

## 3. Discussion

CRS is a rare and usually sporadic disorder, and its cause is still unknown. It compromises various developmental anomalies of the caudal vertebrae, neural tube, hind limbs, urogenital and digestive organs, all of which are derived from the caudal eminence. The severity of the morphologic disorder inversely correlates with residual spinal cord function. The recurrence risk is very small, although it is 200–250 times more frequently in infants of diabetic mothers [3]. 

CRS may exist with no obvious outward signs [4] and, in that case, the diagnosis is often delayed until failed attempts at toilet training bring the child to the attention of a physician [5]. The neurologic manifestations including motor and sensory deficits usually correspond to the level of vertebral agenesis, although in some patients the sensory functions persist below this level [4]. In addition, when these children are evaluated in the newborn or early infancy period, the majority have a perfectly normal neurologic examination. Urodynamic testing, however, will reveal abnormal lower urinary tract function in about one-third of babies younger than 18 months old [6] and it might be the only clue of an underlying neurologic injury involving the lower spinal cord. Urodynamic testing is often characterized by detrusor overactivity, exaggerated sacral refluxes, absence of voluntary control over sphincter function, and detrusor-sphincter dyssynergia (DSD) [5]. This patient manifested DSD with a trabeculated bladder and grade IV reflux. 

CRS is a representative occult spinal dysraphism and as many as 20 % with a neuropathic bladder has renal impairment, which is significantly more frequent than other representative spinal dysraphisms, such as meningomyelocele (MMC) and spinal lipoma (SL). The frequency of renal agenesis in CRS (14.4%) is significantly higher (**P* < 0.01*) than MMC and SL (less than 0.01%). In addition, insufficient bladder voiding is statistically associated with renal damage only in the CRS population. These data suggest that improvement of bladder voiding can lead to a better outcome and earlier intervention is needed in order to preserve residual renal function, although the diagnosis tends to be delayed because of lack of clinical manifestations. CRS seems to represent the population at highest risk, in whom conventional treatment of high residual urine with CIC and antimuscarinic agents could be, at least in some cases, insufficient. More aggressive management of CRS patients, including vesicostomy in selected cases, is suggested [7].

The pathology requiring special attention is orthopedic deformities and bladder and bowel continence, along with preservation of renal function. This helps the child to be independent and socially useful with a better quality of life [1]. Survival is the rule if the vital systems are unaffected or minimally affected. These patients have normal intelligence and therefore lead otherwise normal lives except for neuromuscular deficits of the lower limbs and sphincters; however, secondary neuropathic bladder leading to progressive renal function remains an important comorbid factor. As suggested earlier, CRS patients frequently present with renal impairment, and the lack of outward signs can, at times, result in delayed diagnosis, which leads the patients to exhibit severe progressive renal injury. Earlier detection of the disease and earlier interventions are vital, and urological examinations including urodynamic testing might be the clue of an underlying neurologic injury involving the lower spinal cord.
